# The Hypothalamic-Pituitary-Adrenal Axis: Development, Programming Actions of Hormones, and Maternal-Fetal Interactions

**DOI:** 10.3389/fnbeh.2020.601939

**Published:** 2021-01-13

**Authors:** Julietta A. Sheng, Natalie J. Bales, Sage A. Myers, Anna I. Bautista, Mina Roueinfar, Taben M. Hale, Robert J. Handa

**Affiliations:** ^1^Department of Biomedical Sciences, Colorado State University, Fort Collins, CO, United States; ^2^Department of Basic Medical Sciences, University of Arizona College of Medicine, Phoenix, AZ, United States

**Keywords:** estrogen, androgens, glucocorticoid, sex differences, hypothalamus, development, homeostasis, HPA axis

## Abstract

The hypothalamic-pituitary-adrenal axis is a complex system of neuroendocrine pathways and feedback loops that function to maintain physiological homeostasis. Abnormal development of the hypothalamic-pituitary-adrenal (HPA) axis can further result in long-term alterations in neuropeptide and neurotransmitter synthesis in the central nervous system, as well as glucocorticoid hormone synthesis in the periphery. Together, these changes can potentially lead to a disruption in neuroendocrine, behavioral, autonomic, and metabolic functions in adulthood. In this review, we will discuss the regulation of the HPA axis and its development. We will also examine the maternal-fetal hypothalamic-pituitary-adrenal axis and disruption of the normal fetal environment which becomes a major risk factor for many neurodevelopmental pathologies in adulthood, such as major depressive disorder, anxiety, schizophrenia, and others.

## Introduction

Humans and animals respond to environmental perturbations with a stress response that allows physiological adaptation to the stressor to maintain homeostasis. A major component of the homeostatic response is the hypothalamic-pituitary-adrenal (HPA) axis, an intricate, yet robust, neuroendocrine mechanism that mediates the effects of stressors by regulating numerous physiological processes, such as metabolism, immune responses, and the autonomic nervous system (ANS). The HPA axis consists of a cascade of endocrine pathways that respond to specific negative feedback loops involving the hypothalamus, anterior pituitary gland, and adrenal gland.

There are several critical developmental stages must be attained to ensure proper functionality of the HPA axis and appropriate behavioral and physiological stress-responses in adulthood. The HPA axis begins to develop as early as fetal life and becomes sexually dimorphic during puberty due to differing levels of gonadal hormones. Early life exposure of the offspring to excess fetal glucocorticoid (GC) hormones or environmental perturbations, such as maternal stressors, can alter normal neuropeptide synthesis and lead to a disruption in the development of the HPA axis. This may become detrimental to the fetus later in life as it leads to abnormal physiological function in adulthood, thereby increasing the risk for adult disease.

In this review, we discuss the HPA axis as the central regulator of various physiological responses to stressors. We also examine the activational and organizational effects of hormones during critical periods of development that result in the sexually dimorphic responses of the HPA axis in adults. The effect of environmental perturbations, such as prenatal stress or prenatal exposure to synthetic GC hormones, and the associated susceptibility to stress-related neuropathologies in adulthood are also addressed.

## Components of the HPA Axis

### Morphology and Development of the Paraventricular Nucleus (PVN)

The paraventricular nucleus (PVN) houses three functional neuronal types that act as central regulators of the stress response: parvocellular, neurosecretory magnocellular, and long-projecting neurons. These neurons are characterized by their unique electrophysiological properties (Tasker and Dudek, 1991). Parvocellular neurons display small low threshold depolarizations, while long-projecting neurons generate large low-threshold depolarizations. In contrast, magnocellular neurons do not display low-threshold potentials but are characterized by a distinct return to baseline after depolarizing stimuli (Hernandez et al., 2015; Israel et al., 2016). Further investigation is necessary to identify specific mechanisms that lead to these unique properties.

*Neurosecretory parvocellular* neurons send their axons to the external zone of the median eminence to regulate the secretion of releasing factors [e.g., corticotropin-releasing hormone (CRH), thyrotropin-releasing hormone (TRH)] into the hypothalamohypophyseal portal vasculature to control the secretion of corresponding anterior pituitary hormones. An *anterior* parvocellular division extends from the rostral boundary of the PVN to the rostral boundary of the medial magnocellular division, just lateral to the periventricular area. The *medial* parvocellular division lies lateral to the periventricular area and medial to the medial magnocellular division. Neurons in the anterior and medial parvocellular groups project to the median eminence or other hypothalamic and extrahypothalamic regions.

*Neurosecretory magnocellular* neurons project to the neurohypophysis to regulate the secretion of oxytocin (OT) and arginine vasopressin (AVP) directly into the general circulation (Vandesande and Dierickx, 1975; Rhodes et al., 1981; Swanson and Sawchenko, 1983). The rat is the most studied species for characterizing PVN morphology and in the rat PVN, magnocellular neurons distribute into two distinct areas. The *medial* magnocellular division lies anteromedially within the PVN and contains mostly OT expressing neurons. The *lateral* magnocellular division is largely comprised of AVP-expressing neurons that are surrounded by a loop of OT neurons. *Long-projecting* neurons send their axons to the brainstem and spinal cord regions to control autonomic and somatosensory function.

The hypothalamus is derived from the anteroventral neuroectoderm during early development (Takata et al., 2017). Mapping of gene expression along the rostral-caudal axis shows that the early hypothalamic primordium differentiates into the floor plate, basal plate, and alar plate. The dorsal-most portion of the alar plate gives rise to the PVN and supraoptic nucleus (SON) and is identified by the expression of *Brn-2, Otp, and Sim1* genes and the absence of *Dlx*, *Arx*, *Gad67*, *Isl1*, and *Vax1* genes that are found in the subregion immediately below the PVN (Morales-Delgado et al., 2014).

The transcription factor, *Brn-2* (POU-homeodomain protein BRIN-2), is endogenously expressed in both parvocellular and magnocellular neurons (He et al., 1989). *Brn-2* null mutations in rodents demonstrate a failure to differentiate between CRH parvocellular neurons and OT and AVP magnocellular neurons, suggesting it is necessary for terminal differentiation of these hypothalamic cells (Schonemann et al., 1995).

The homeobox gene, *Otp*, transcribes a transcription factor that helps regulate differentiation and maturation of the neurosecretory PVN neurons expressing TRH, AVP, and OT. In mice, the induction of a missense mutation in the *Otp* gene causes acute onset obesity and increased anxiety, phenotypes that have similarly been shown to be modulated by AVP and OT. Moreover, *Otp* seems to be necessary for regulating the transcriptional activity of PVN neurons (Moir et al., 2017).

The *Sim1* transcription factor acts as another key regulatory gene of the PVN, encoding a protein that also regulates AVP, TRH, and OT expression, as well as CRH and somatostatin (Michaud et al., 1998).* Sim1* knock-out mice show severe loss of AVP, TRH, CRH, OT, and somatostatin neurons and rarely survive to adulthood (Michaud, 2001), while heterozygous mice display early obesity, hyperinsulinemia, hyperphagia, and hyperleptinemia, phenotypes that are associated with PVN neurosecretory neurons (Michaud, 2001). The Sim1 protein has been shown to dimerize with Aryl hydrocarbon receptor nuclear translocator 2 (ARNT2), which is thought to differentiate PVN and SON neurons. *Brn2*, a downstream target of the Sim1/ARNT2 dimer, also mediates Sim1 function. *Brn2* promotes the expression of AVP, OT, and CRH in the PVN, and decreased numbers of these cell phenotypes are seen in *Brn2* knock-out mice (Schonemann et al., 1995).

### Morphology and Development of the Pituitary Gland

The pituitary gland functions largely in response to releasing factors from the hypothalamus. The pituitary gland is divided into two structures: the adenohypophysis (anterior pituitary) and the neurohypophysis (posterior pituitary; Dorton, 2000). The adenohypophysis constitutes 80% of the pituitary gland and houses specialized hormone-producing cells that synthesize and secrete several hormones, including, but not limited to, growth hormone (GH), thyroid-stimulating hormone (TSH), follicle-stimulating hormone (FSH), luteinizing hormone (LH), prolactin and adrenocorticotropic hormone (ACTH). These hormones target various types of tissues to mediate several physiological processes in response to stress (Scanes, 2015). Annexin-1 (formerly known as lipocortin 1) is another protein that exists in the anterior pituitary. While it is not directly involved in the synthesis of hormones discussed above, it is an important regulator of their secretion through inhibitory pathways (for a more detailed description of the actions of annexin-1, see reviews by John et al., 2004; Denef, 2008).

The adenohypophysis can be further divided into the pars distalis, pars intermedia, and the pars tuberalis. The pars distalis is composed of chromaffin and chromophobe cells and is where most hormone synthesis occurs. The pars tuberalis is an extension of the pars distalis and houses epithelial cells and the hypophyseal portal vessels that connect the anterior pituitary to the hypothalamus. The pars intermedia, located between the pars distalis and neurohypophysis, secretes products produced by the proopiomelanocortin (POMC) gene, particularly melanocyte-stimulating hormone (MSH; Ilahi and Ilahi, 2020).

In contrast to the adenohypophysis, the neurohypophysis is directly connected to the hypothalamus by axonal projections of magnocellular neurons originating from either the PVN or SON. The posterior pituitary stores OT and AVP synthesized by these neurons and secretes them into the general circulation in response to various hypothalamic releasing factors. OT is required for lactation, while AVP is involved in the regulation of osmotic balance (Borrow et al., 2019). Peptide hormones synthesized in the SON and PVN travel along axons to their terminals in the posterior pituitary where they are released into the general circulation in response to signals from their hypothalamic cell bodies (Ohbuchi et al., 2015).

The development of the pituitary gland is complex, yet unique because of its dual origin.

In humans, during the fourth week of gestation, cells of the oral portion of the ectoderm begin to thicken to form the hypophyseal placode (Ericson et al., 1998). The hypophyseal placode elongates to form Rathke’s pouch. At 6–8 weeks of development, the base of Rathke’s pouch is separated from the oral epithelium. The rapid proliferation of cells of the anterior wall of the pouch forms the anterior lobe of the pituitary (pars distalis) and the slower proliferation of cells of the posterior wall give rise to the intermediate lobe or pars intermedia. By contrast, a specific portion of the ventral diencephalon located dorsal to Rathke’s pouch gives rise to the infundibulum from which posterior pituitary originates (Amar and Weiss, 2003; Zhu et al., 2007).

The development of the pituitary gland is mediated by several cellular transcription factors. *Sonic hedgehog*, expressed in the oral ectoderm, and *bone morphogenic protein 4*, and *fibroblast growth factor 8*, found in the ventral diencephalon, are all important signaling genes that initiate cellular proliferation of pituitary cells. These genes have also been shown to affect the expression of transcription factors that contribute to the differentiation of specific pituitary lineages, however specific mechanisms are not yet known (Ericson et al., 1998). Furthermore, the transcription factor, *Tpit*, is critical for the expression of the POMC gene. POMC is expressed in corticotrophs, the first pituitary cell type to terminally differentiate (about gestation day 12.5 in the mouse; Pulichino et al., 2003). A deficiency of *Tpit* blocks terminal differentiation, but not a commitment to the corticotroph lineage (Pulichino et al., 2003). Somatotrophs, lactotrophs, and thyrotrophs are differentiated through the influence of transcription factors, *Prop1* and *Pit-1 whereas* gonadotrophs require* GATA-2* and* SF1* signaling molecules for terminal differentiation. Although these terminal cell lineages are found in the pituitary gland, the existence of a common ancestral precursor pool is unclear (Zhao et al., 2001).

### Morphology and Development of the Adrenal Gland

The adrenal gland of adult mammals is surrounded by a fibrous capsule and is composed of two regions with differing embryological origins (Bornstein et al., 1991). While the adrenal medulla, responsible for catecholamine production, derives from neuroectoderm, the steroid-hormone producing adrenal cortex has an embryonic origin from the adrenogonadal primordium (Sun et al., 2018).

The adrenal medulla is composed of chromaffin cells that secrete epinephrine and norepinephrine following sympathetic stimulation. They can be considered as a grouping of modified postganglionic neurons that are directly innervated by preganglionic neurons from the central nervous system (CNS; McCorry, 2007). Thus, the adrenal medulla is an important component of the ANS and responds very rapidly to stressors, releasing epinephrine and norepinephrine into the bloodstream to affect heart rate, blood pressure, metabolism, and others (Vinson et al., 1994). These hormones are classically involved in the “fight or flight” response. The effects of epinephrine and norepinephrine on various physiological systems are emphasized by changes noted in patients with pheochromocytoma, a catecholamine secreting neuroendocrine tumor of the adrenal chromaffin cells (Pacak, 2011). Symptoms include sweating, heart palpitations, markedly elevated blood pressure, nausea, tremors, and weight loss (Parenti et al., 2012).

Histologically, the adrenal cortex is composed of three zones. The outer zona glomerulosa produces aldosterone, which is involved in water and mineral balance through its actions on the kidney and colon (Rakova et al., 2017). The intermediate zona fasciculata is the thickest region of the adrenal cortex and synthesizes corticosteroids (primarily cortisol in the human, corticosterone in most rodents) and androgens. Similarly, the innermost zona reticularis also synthesizes adrenal androgens (Longcope, 1986). Of note, dehydroepiandrosterone (DHEA) is the most abundant circulating adrenal androgen in adult humans, whereas these are very low in adult rats and mice (Dumontet et al., 2019). Both the zona reticularis and fasciculata are regulated by ACTH and in the absence of ACTH, these zones atrophy, whereas following chronic ACTH stimulation, these zones hypertrophy (Gallo-Payet et al., 2017).

The adrenal cortex is derived from mesoderm and is dependent upon several transcription factors such as SF-1 (*steroidogenic factor-1*) and DAX-1 (*dosage-sensitive sex reversal-adrenal hypoplasia)*. Deletion of either of these genes results in the absence of adrenocortical development in mice (Hammer et al., 2005). During human gestation, an inner fetal adrenal zone makes up the bulk of the adrenal gland, and an adult-like “definitive” zone, a group of small tightly packed cells is also present (Coulter, 2004). The human fetal adrenal responds to ACTH, but because of the absence of the 3-hydroxysteroid dehydrogenase enzyme, the fetal adrenal mainly produces DHEA and DHEA sulfate (Ishimoto and Jaffe, 2011). These fetal adrenal steroids serve as precursors of maternal placental estrogens. The definitive zone is the major producer of fetal cortisol in response to ACTH stimulation.

By contrast, the developing rodent adrenal is quiescent. It is questionable whether the rodent adrenal contains a fetal adrenal zone *per se*, although some studies indicate a transient fetal adrenal zone based on the presence of fetal adrenal enhancer elements (Zubair et al., 2006). While the adult cortex of rodents increases in size from late gestation through puberty, the fetal zone cells disappear gradually and accumulate along the boundary with the adrenal medulla (Morohashi and Zubair, 2011). However, even after the adult zones are developed, the adrenal gland of the rodent fetus does not yet express aldosterone synthase nor does it respond to stimulation by increasing mineralocorticoid or GC synthesis (Ehrhart-Bornstein et al., 1998).

## The Physiology of Stress and Central Regulation of the HPA Axis

**Figure 1:**
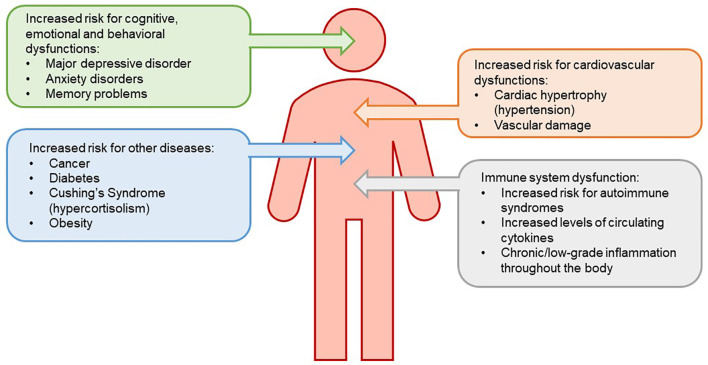
Chronic stress leads to reduced sensitivity of the negative feedback system that governs the hypothalamic-pituitary-adrenal (HPA) axis. The loss of this negative feedback system is due to an increased level of circulating glucocorticoids (GCs). HPA axis dysregulation results in downstream physiological consequences, increasing risk for immune system dysfunction, mood disorders, metabolic disease, and cardiovascular disease.

It is well established that animals and humans respond to threats to their welfare by activating neurons that control neuroendocrine and autonomic responses. For the HPA axis, the endocrine response is characterized by the secretion of GCs from the adrenal cortex. Circulating GCs act on a variety of tissues to mobilize energy stores, induce lipolysis and proteolysis, potentiate vasoconstriction driven by the ANS, suppress reproduction, and alter stress-related behaviors, to allow homeostasis (Papadimitriou and Priftis, 2009). Most of the physiological responses to acute elevations in GCs that occur following stressors, such as enhanced cognition and metabolism and inhibition of immune function must be beneficial, as they permit the “fight or flight” response. By contrast, although some benefits to chronic stress exist, chronic activation of the HPA axis has deleterious effects on immune, cardiovascular, metabolic, and neural functions and may decrease the resilience of neurons and glia to subsequent insults (McEwen, 1998; Jauregui-Huerta et al., 2010; Heck et al., 2020), resulting in increased risk for several diseases (see Figure 1 for summary describing effects of chronic stress on health conditions). Whether these are direct or indirect effects of glucocorticoids remains to be determined.

### Negative Feedback Circuitry

The HPA axis is governed by a closed-loop GC-dependent negative feedback system that is essential for the termination of the stress response. Normal HPA function is highly influenced by the dose and duration of GC exposure (Abe and Critchlow, 1980; Sapolsky et al., 2000). For example, adrenalectomy decreases GC secretion, which increases PVN neuropeptide expression and secretion in both basal and stress-induced states (Sawchenko, 1987; Imaki et al., 1991). Negative feedback can also act at the level of the PVN, the anterior pituitary, and indirectly *via* brain regions that project to the PVN (Akana et al., 1986; Sawchenko, 1987; Bradbury et al., 1994). Notably, primary sites of negative feedback differ between endogenous and synthetic GC. Endogenous GCs, such as corticosterone, primarily induce negative feedback at the level of the PVN, while the synthetic GC, dexamethasone (DEX), functions as a glucocorticoid receptor (GR) agonist to inhibit GC release at the level of the anterior pituitary gland (Spiga et al., 2017).

GC-dependent negative feedback has further been shown to rely on the rhythmic release of GC in diurnal and ultradian patterns that are fundamental to the termination of the stress response (Sapolsky et al., 2000; Gjerstad et al., 2018). In all vertebrates, a peak of circulating corticosterone occurs just before the onset of daily activity. The diurnal rhythm of HPA axis activity is driven by the central circadian pacemaker, the suprachiasmatic nucleus (SCN), which has major inputs to the PVN to regulate daily rhythms of GC output (Lightman and Conway-Campbell, 2010). An ultradian variation in GC secretion is composed of the pulsatile release of corticosterone and ACTH that occur in response to differential timing of the stimulus and feedback signals within the HPA axis (Walker et al., 2012). In contrast to the circadian rhythm, the ultradian rhythm is not under central regulation of the SCN but is likely generated by a pituitary-adrenal feed-forward—feedback loop under constant CRH infusion. Moreover, when high levels of CRH were infused, the ultradian pattern was disrupted and corticosterone oscillations were dampened, further suggesting a dose-dependent effect of CRH on the patterns of GC secretion (Rankin et al., 2012).

Since circulating GCs can bind to both GR and mineralocorticoid receptor (MR), both receptors are involved in the negative feedback regulation of the HPA axis. MRs have a greater affinity for corticosterone (cortisol in humans; Reul and de Kloet, 1985) and consequently, they are predominantly bound during low or basal secretion of corticosteroid (Reul and de Kloet, 1985). Adrenalectomy increases basal CRH and ACTH levels, suggesting that a decrease in circulating corticosterone removes the negative feedback signal (Dallman et al., 1985, 1987; Rabadan-Diehl et al., 1997) whereas corticosterone replacement at doses that bind MR selectively, returns ACTH levels to baseline (Bradbury et al., 1994). Moreover, the hippocampus (HC), a primary site for HPA regulation during basal states, expresses MR at high levels. MR antagonists administered directly to the rat HC elevate basal ACTH and corticosterone levels like those seen after adrenalectomy (Van Haarst et al., 1997). Studies using transgenic mice that overexpress forebrain MR show reductions in the corticosterone response to restraint and decreases in anxiety-like behaviors (Rozeboom et al., 2007). Together, such data suggest that the ratio of MR:GR are as influential as absolute levels for regulating stress-induced HPA axis activity and stress-related behaviors.

In contrast to MR, GR has a lesser affinity for corticosterone and is thought to be the primary target for negative feedback when GC levels are elevated (Ruel and de Kloet, 1985). GRs remain mostly unoccupied during the basal state but are quickly occupied after a stress-induced increase in circulating GCs (Reul and de Kloet, 1985). This supports the hypothesis that GR activation the return of HPA activity to baseline following high amplitude secretion of corticosteroids after an acute stressor. Like MR, GR is highly expressed in the HC, as well as in the PVN and adenohypophysis (Ahima and Harlan, 1990; Morimoto et al., 1996). Initiation of HPA axis negative feedback occurs *via* GR expressing neurons in the HC (Sapolsky et al., 1984) and the hypothalamus (Weidenfeld et al., 2002), following stress-induced elevations in corticosterone. At the PVN, this first acts upon AVP neurons (Kovács et al., 2000). The importance of GR in negative feedback regulation is further demonstrated using a transgenic mouse model. Selective knockout of forebrain GR causes an increase in basal and stress-induced corticosterone levels (Kolber and Muglia, 2009), further implicating GC binding sites in the forebrain. In comparison, Wei et al. (2004) showed that the overexpression of forebrain GR does not alter basal ACTH or corticosterone levels, indicating that the forebrain GR is not the only player in regulating the baseline activity of the HPA axis. Selective disruption of GR in the PVN increased CRH immunoreactivity in the PVN, with corresponding increases in levels of plasma ACTH and corticosterone, supporting the hypothesis that GR is involved in negative feedback (Solomon et al., 2015). Furthermore, GR has been reported to be absent in the SCN, suggesting an alternate circadian-like feedback mechanism where GC influences HPA axis activity as discussed previously.

### Regulation of CRH in the Brain and Pituitary

CRH is abundantly produced in neurons of the PVN as well as other brain areas and is highly conserved between humans, rats, and mice (Wamsteeker-Cusulin et al., 2013). While the predominant site of CRH expression is the PVN, CRH is expressed in other brain areas including the central n. of the amygdala (AMY; CeA), the bed n. of the stria terminalis (BNST), and the cortex and HC. It is well-known that stressors can similarly increase *crh* expression in the PVN and CeA (Herman and Tasker, 2016) with increases in the primary transcript (heterologous nuclear RNA) for *crh*, rising within minutes following the application of a stressor (Evans et al., 2013). This is followed by subtler increases in *crh* mRNA (Vazquez et al., 2006). Following enhanced cellular activity such as following a kainic acid-induced seizure, *crh* expression also increases and numerous CRH-*ir* neurons can be visualized in brain areas that normally express modest levels of CRH, including the HC, BNST, and globus pallidus (Foradori et al., 2007). Within the PVN, CRH is expressed by both pre-autonomic neurons that project to the brainstem and spinal cord (Swanson and Kuypers, 1980), as well as neuroendocrine neurons that project to the median eminence.

The actions of CRH are mediated by two receptor types, CRF-R1 and CRF-R2. The cloning and characterization of the CRH receptor were originally reported by Chen et al. (1993). This receptor-bound CRH with high affinity and selectivity and was coupled to adenylate cyclase to increase intracellular cyclic adenosine monophosphate (cAMP). Subsequently, a second CRH receptor, CRF-R2, was found to possess approximately 70% homology to CRF-R1. CRF-R2 had a different distribution in the brain, being found in subcortical regions, with the greatest expression in the lateral septum and ventromedial n. (VMN), in contrast to the CRF-R1 which was found to be highly expressed in neocortical and cerebellar regions. In the adenohypophysis, CRF-R1 was expressed at very high levels whereas CRF-R2 was expressed at much lower levels (Chalmers et al., 1995). Such studies indicate widespread effects of CRH with physiological responses selectively mediated by two different receptors.

CRH action at the pituitary is primarily mediated by CRF-R1, yet, its actions can be modified by a CRH binding protein (CRH-BP) which is highly expressed by both pituitary and hypothalamus, although only at low levels in the PVN (Chalmers et al., 1995). CRH-BP is found in cells expressing CRH receptors and in plasma and rapidly binds CRH with high affinity, thereby acting as a competitive inhibitor of CRH actions (Kalin, 2018). A sex difference in CRH-BP mRNA has been shown in the mouse pituitary with females having much greater levels than males (Speert et al., 2002). Moreover, stress increased CRH-BP mRNA and protein expression in POMC neurons in females more than males. Whereas data show that CRH-BP deficient mice exhibit elevated corticosterone levels (Speert et al., 2002), indicating that CRH-BP is functionally involved in preventing the actions of CRH to reduce the HPA response to stress, this is inconsistent with the greater basal and stress-induced levels of plasma corticosterone in females, suggesting other mechanisms are also involved in setting up sex differences in HPA function.

### CRH as a Regulator of ACTH

ACTH is synthesized from high molecular weight (266 amino acids) precursor protein, POMC, in the anterior pituitary (Harno et al., 2018). The POMC gene consists of three exons and two introns. All functional peptide products of the POMC gene are encoded in exon 3 including N-terminal glycopeptide, Υ-MSH, ACTH, α-MSH, CLIP, β-lipoprotein, Υ-lipoprotein, β-MSH, and β-endorphin. Their evolutionary importance is indicated by the fact that the regions encoding α-MSH, ACTH, and β−endorphin are highly homologous between mammalian species including humans. Consistent with the important role of ACTH in mediating the neuroendocrine stress response, POMC mRNA levels in the anterior pituitary are upregulated by CRH and inhibited by GCs (Deng et al., 2015, 2017). By contrast, in the intermediate lobe, there is no effect of GCs on POMC mRNA levels due to the very low expression of GR (Wang et al., 2015).

### AVP and OT as a Regulator of ACTH

While CRH is the primary regulator of ACTH secretion by the adenohypophysis, supporting roles of AVP and OT as co-secretagogues have also been shown (Herman et al., 1992). Although AVP has been described as regulating osmotic balance, and OT as a principal hormone for parturition, both neuropeptides are co-expressed in about half of the parvocellular CRH-expressing neurons of the PVN after adrenalectomy (Sawchenko et al., 1984). Both are thought to be co-released with CRH (Bondy et al., 1989; Raadsheer et al., 1993) to potentiate CRH’s secretagogue activity at the corticotroph (Rivier and Vale, 1983; Schlosser et al., 1994). Moreover, both AVP and OT can stimulate ACTH secretion even in the absence of CRH (Gillies et al., 1982; Schlosser et al., 1994) by activating the V1b receptor on corticotrophs (Schlosser et al., 1994). In contrast, when applied to the PVN, or following intracerebroventricular injection, OT and AVP inhibit HPA axis responses (Neumann et al., 2000; Landgraf and Neumann, 2004). It has also been shown that these neuropeptides modify PVN function in a paracrine fashion through local dendritic release within the PVN (Neumann, 2007), the net result being regulatory effects on the PVN that are very different from actions on the adenohypophysis.

In the adenohypophysis, AVP is hypothesized to also arise by collateral projections to the median eminence from magnocellular neurons that target the posterior pituitary, or through vessels from the posterior pituitary that connect to the adenohypophysis. Interestingly, the mouse does not seem to co-express AVP in CRH neurons to the same extent as the rat (Biag et al., 2012) suggesting that co-release with *crh* may be minimal in the mouse. Nonetheless, AVP drives the rapid release of ACTH from anterior pituitary corticotrophs, although, by itself, AVP alone causes only a small ACTH response. However, AVP potentiates CRH-driven ACTH release *in vivo* and *in vitro*, when cells are first exposed to CRH. In contrast, if cells are first exposed to AVP, CRH does not potentiate AVP-stimulated ACTH secretion (Roper et al., 2011). Thus, it appears that AVP and CRH can cooperate, but not substitute for each other to regulate ACTH secretion.

## Neuroendocrine Stress Responses and Central Inputs

To cope with the physiological changes following a stressor, parvocellular neurons of the PVN integrate neural or hormonal input from a variety of sources leading to a physiologic and metabolic response. Direct inputs arising from the brainstem are essential to integrating HPA reactions to systemic stressors. Projections originating from noradrenergic and adrenergic neurons in the nucleus of the solitary tract (NTS), locus coeruleus, and the ventrolateral medulla that innervate the parvocellular PVN have been identified (Kítazawa et al., 1987; Hornby and Piekut, 1989; Cunningham et al., 1990). CRH neurons of the medial parvocellular PVN receive noradrenergic innervation from A2 adrenergic cell groups of the NTS (Kítazawa et al., 1987; Hornby and Piekut, 1989). The co-expression of alpha(1) and alpha(2) receptors in medial parvocellular CRH neurons (Cummings and Seybold, 1988; Day et al., 1997, 1999) allows norepinephrine to rapidly increase *crh* mRNA (Itoi et al., 1999; Cole and Sawchenko, 2002; Khan et al., 2007). Alpha(1) adrenergic receptors may be primarily responsible for the stimulatory effects of norepinephrine (Cummings and Seybold, 1988; Kiss and Aguilera, 1992; Khan et al., 2007), whereas alpha(2) adrenergic receptors, specifically alpha(2A) and alpha(2C), may be essential in inhibition of norepinephrine release at the presynaptic membrane (Bucheler et al., 2002).

Another circuit that strongly influences HPA responses to stress are projections from the median and dorsal raphe nuclei. Serotonergic fibers to the parvocellular PVN (Sawchenko et al., 1983; Zhang and Fogel, 2002) stimulate the HPA axis (Van de Kar and Blair, 1999). Serotonin (5-HT) 2C receptors have been implicated in 5-HT-induced activation of the HPA axis (Heisler et al., 2007). However, 5-HT1A receptors have also been shown to increase ACTH secretion (Rossi et al., 2010) and knockout of 5-HT1b receptors causes a 50% reduction in the diurnal rise in plasma corticosterone (Sollars et al., 2014). Restraint-induced elevations in ACTH and corticosterone can be increased by blocking 5-HT7 receptors (García-Iglesias et al., 2013), while 5-HT can inhibit GABAergic synaptic transmission at the PVN (Lee et al., 2008) providing evidence that the effect of 5-HT on PVN neurons vary depending on where the afferents terminate.

### Limbic Pathways Regulate CRH Neuron Function

Important information to PVN neurons also arises from a variety of limbic structures, including the BNST, a group of related subnuclei that directly project to the parvocellular PVN (Cullinan et al., 1993; Dong et al., 2001). BNST neurons express androgen receptor (AR) and estrogen receptor (ER; Simerly, 1993), and play a crucial role in gonadal steroid regulation of HPA function. The majority of these neurons are GABAergic (Cullinan et al., 1993) and their activity can be enhanced by collateral CRH afferents (Kash and Winder, 2006; Dabrowska et al., 2011). The BNST also contains CRH neurons that project to the PVN (Dong et al., 2001; Dong and Swanson, 2006) and contain ARs (Heck and Handa, 2019a). Lesion studies show that BNST GABAergic neurons inhibit CRH mRNA levels, and inhibit the corticosterone responses to stress (Dunn, 1987; Herman et al., 1994) as does treatment with androgens (Lund et al., 2005). However, not all BNST neurons are inhibitory since selective lesions of the anterior or lateral BNST can decrease ACTH secretion (Gray et al., 1993; Herman et al., 1994). Nonetheless, the BNST represents an important modulating region that is gonadal steroid hormone-sensitive.

Other limbic regions known to modulate stress-responsive HPA axis activity include the HC, prefrontal cortex (PFC), CeA, medial AMY (MeA), and lateral septum. These regions do not directly innervate parvocellular PVN but relay through areas such as the BNST or peri-PVN (Dong et al., 2001; McKlveen et al., 2015). The predominantly glutamatergic projections from HC and PFC (Walaas and Fonnum, 1980) are translated to inhibitory actions on the HPA axis *via* the GABAergic nature of these relays (Diorio et al., 1993; Herman et al., 1998; Figueiredo et al., 2003). By contrast, AMY to BNST and peri-PVN projections are largely GABAergic. Thus, reducing the inhibitory tone is an effective mechanism to increase HPA axis activity (Swanson and Petrovich, 1998).

## Development of Neuropeptides and Their Regulation

### Development of CRH

In rats, *Crh* mRNA is detected as early as embryonic day 17 in the PVN (Grino et al., 1989), while the peptide is found to be expressed the following embryonic day 18 (Bugnon et al., 1982). In contrast to the CRH peptide, *Crh* mRNA is robustly expressed during embryonic days 18 and 19, followed by a reduction on days 20 and 21. CRH finally reaches adult levels of expression by the end of postnatal day (PND) 7 (Grino et al., 1989). In the mouse hypothalamus, CRH expression is detected initially on embryonic day 13, but mimics the same trend as the rat and decreases just before the time of birth (embryonic day 17–18 in mice), followed by an increase to adult levels thereafter (Schmidt et al., 2003).

### Development of AVP and OT

In all species examined, AVP mRNA has been detected during development before OT mRNA, and in greater quantities (Wolf et al., 1984). In rodent studies, immunohistochemistry (Whitnall et al., 1985), quantitative PCR (Lipari et al., 2001), quantitative immunoassay (Sinding et al., 1980), and *in situ* hybridization assays (Laurent et al., 1989), show that AVP is consistently found in the developing fetus’ brain, while OT is not significantly expressed until 1–2 days following parturition (Yamashita et al., 1988; Laurent et al., 1989). Interestingly, more recently, Tamborski et al. (2016) detected OT mRNA as early as embryonic day 16 in females, but not until PND 2 in males, indicating a sex difference in the development of central oxytocin that may contribute to sex differences in adulthood. The AVP circuitry also becomes sexually dimorphic with the greater synthesis of AVP in males than females during early postnatal stages (for more details, see reviews: Szot and Dorsa, 1993; Rood and De Vries, 2011; Rood et al., 2013). In adulthood, many AVP populations of neurons are androgen-responsive (de Vries and Al-Shamma, 1990). Both AVP and OT expressing-neurons continue to increase postnatally in the PVN, and SON of the hypothalamus (Van Tol et al., 1987; Almazan et al., 1989; for further details of OT and AVP development, see reviews: di Scala-Guenot et al., 1990; Bales and Perkeybile, 2012).

## Neurodevelopment of the HPA Axis

### Estrogens and Androgens Have Activational Actions on the HPA Axis

Gonadal steroids are crucial hormones in the regulation of the adult HPA axis, resulting in stark differences in responsiveness of the axis between sexes. Studies show that testosterone generally depresses the stress response (Viau and Meaney, 1996) while estradiol can either enhance or inhibit it (Handa et al., 2009; Zuloaga et al., 2014). The actions of estrogens are controlled by two major types of ER, ERα and ERβ. ERs are ligand-activated transcription factors that bind to estrogen response elements (ERE) in gene promoters, thereby providing a link between gonadal hormones and transcriptional responses of the HPA axis (McEwen et al., 1999). ERα and ERβ both have unique yet overlapping expression patterns in the brain. ERα is highest in the VMN, arcuate n. (ARC) and medial preoptic area (MPOA), whereas ERβ is highly expressed in the PVN, SCN, and SON (in the rat). Overlapping brain regions containing both ERs include BNST, MeA, and MPOA (Hiroi et al., 2013). The unique expression of ERα and ERβ accounts for various physiological functions, specifically the HPA response to acute stress considering that EREs exist upstream of AVP, OT, and CRH gene promoters and can directly regulate gene transcription (Shapiro et al., 2000; Miller et al., 2004; Pak et al., 2007; Hiroi et al., 2013).

Estrogens have been shown to augment HPA axis activity and the release of the stress-related secretagogues at several sites due to the broad expression of ERs. In the adrenal gland, estradiol increases the adrenal response to ACTH administration (Patchev et al., 1996). Similarly, reports have shown that at the level of the adenohypophysis, estradiol results in a greater response to CRH demonstrated by increased ACTH secretion. Ovariectomy decreases HPA axis stress-responses and these effects can be reversed by replacement of estradiol to females (Seale et al., 2004). Increased PVN CRH immunoreactivity following estradiol treatment has also been shown (Patchev et al., 1996), with more recent studies displaying an overlap of ERβ expression in CRH neurons of the mouse PVN, further suggesting a possible mechanism for augmentation of HPA axis function by estrogens (Oyola et al., 2017). By contrast, some studies have demonstrated that estradiol decreases HPA axis activity or has no effect (Young et al., 2001; Ochedalski et al., 2007). Treatment with estradiol decreased neuronal activation in the PVN (Figueiredo et al., 2007), as well as reduced ACTH secretion (Young et al., 2001) and expression of *Crh* (Ochedalski et al., 2007). Varying reports of the effects of estradiol on the HPA axis could be explained by differing experimental conditions, such as a dose or duration-dependent effect (Figueiredo et al., 2007). Opposing effects of estradiol may also occur due to different signaling mechanisms of ERα and ERβ (Tsigos and Chrousos, 2002). In ovariectomized female rats, ERα agonist, propylpyrazoletriol, increases stress-induced GC secretion while diarylpropionitrile, an ERβ agonist, decreases stress-induced GC secretion (Weiser and Handa, 2009). It has also been proposed that ERα works by reducing the inhibitory tone of GABAergic neurons that project to the PVN, such as those in the BNST, hippocampus, and peri-PVN (Handa and Weiser, 2014) but has limited expression in PVN-specific neurons (Oyola et al., 2017), implicating indirect effects on the HPA axis (Weiser and Handa, 2009). Meanwhile, ERβ is proposed to directly alter HPA axis function since it is found to be co-expressed with several PVN neuropeptides, such as CRH, OT, and AVP (Lund et al., 2006; Oyola et al., 2017).

Androgens are consistently reported to inhibit HPA axis activation and activity (Rosinger et al., 2018; Heck and Handa, 2019a,b). Castration of male rodents removes endogenous androgens, increasing stress-induced secretion of ACTH and corticosterone (Handa et al., 2013). Further, testosterone or dihydrotestosterone replacement is consistently shown to reverse the inhibitory effects, reducing the ACTH and corticosterone response to an acute stressor, suggesting the inhibitory role of testosterone on the HPA axis (Williamson and Viau, 2008). The alterations in ACTH and corticosterone were not accompanied by alterations to CRH sensitivity in the pituitary, suggesting a more central-mediated effect of androgens. Recent studies (Seale et al., 2005b) show that the HPA axis is inhibited by testosterone directly through ARs or indirectly through metabolism with other co-regulatory elements that bind ARs or ERs.

Notably, the effects of dihydrotestosterone, a potent androgenic metabolite of testosterone, are important in the suppression of the HPA axis and GC secretion following stress. Central administration of a 5α-reductase inhibitor, finasteride, blunted the effects of testosterone, and not dihydrotestosterone, implicating a central role for the enzyme, 5α-reductase, in the reduction of testosterone to dihydrotestosterone in HPA reactivity to stress (Handa et al., 2013). Consistent with a study by Handa et al. (2013), dihydrotestosterone injected near the PVN in a gonadectomized, adult male decreases ACTH and corticosterone secretion (Lund et al., 2006). Androgens additionally are reported to decrease CRH response to stress in castrated males, indicating indirect action of testosterone on CRH synthesis since ARs are not expressed by hypophysiotropic CRH neurons (Bingaman et al., 1994). In rodent studies, dihydrotestosterone is metabolized intracellularly to 5α-androstane 3β, 17β-diol (3β-diol), which subsequently binds ERβ to inhibit the HPA axis. Thus, although interactions between androgen and estrogen actions can occur at the level of ligand identity, interactions on a molecular level are harder to identify. Recently, Mahfouz et al. (2016) showed a significant overlap between AR and ER in MPOA and hippocampus in rodents, using genome-wide spatial co-expression of AR and ER targets. Whether this overlap indicates functional interactions at a cellular level in rodents remains to be determined, with the caveat that additional factors may modify these cellular mechanisms in humans.

It has been proposed that sex differences arise, in part, due to varying levels of GR and MR and the availability of corticosteroids in the brain. Corticosteroid-binding globulin (CBG), a glycoprotein produced by the liver, binds to circulating corticosterone. CBG enhances corticosteroid stability during transport to target tissues, but it also prevents corticosteroids from binding to GR or MR (de Kloet et al., 2005). Only free corticosterone can exert physiological effects through its actions on its receptors. Thus, it is important to make a distinction when comparing total plasma corticosterone levels and available corticosterone. In females, CBG is found at levels that are 2-fold higher than in males, but levels of total corticosterone are also significantly higher. Therefore, the increased levels of CBG may help buffer the increased amount of total plasma corticosterone, contributing to the lack of a sex difference in free corticosterone levels (McCormick et al., 2002). Moreover, CBG binds acute stress-induced corticosterone, resulting in a delayed free corticosterone response in comparison to total plasma corticosterone, and implicating CBG as an important buffer for available corticosteroids (Qian et al., 2011). The greater levels of CBG in females likely contributes to the increased HPA axis activity when compared to males, whereas, low levels of CBG in males may lead to higher availability of free corticosterone and a more robust negative feedback on the HPA axis (Viau and Meaney, 1996; Tinnikov, 1999).

### Evidence for Organizational Actions of Gonadal Steroids on HPA Axis

**Figure 2:**
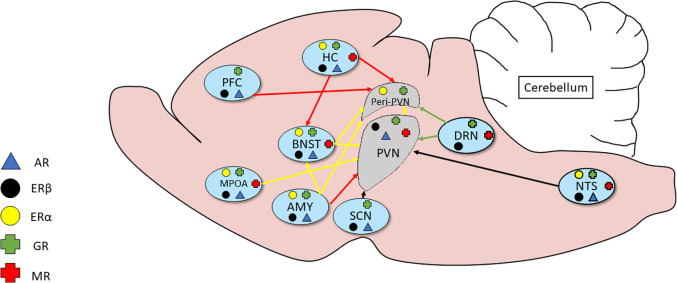
Relationship of inputs of adrenal and gonadal steroid hormone receptors to the circuitry of the HPA axis. Several brain regions secrete adrenal and gonadal hormones that act on receptors in the peri-PVN and paraventricular nucleus (PVN). Much of the expression shows considerable overlap.

The sex differences in adult stress responses may also be programmed by neonatal exposure to gonadal steroids suggesting an organizational effect on the neural circuitry controlling patterns of corticosteroid secretion (Seale et al., 2005a,b) and shaping brain morphology in some brain sites controlling HPA axis function in adulthood (Green and McCormick, 2016). The mechanism(s) by which gonadal steroid hormones act to influence HPA function has not been completely resolved but evidence for androgens and estrogens modulating adrenal (Kitay, 1965), pituitary (Coyne and Kitay, 1969; Viau and Meaney, 2004) and hypothalamic functions (Handa et al., 1994; Viau and Meaney, 1996; Viau et al., 2001) has been reported. The considerable overlap in gonadal and adrenal steroid hormone receptor expression within the neural circuitry of the PVN supports this as a mechanism (Figure 2; see review Goel et al., 2014 for a more in-depth overview).

There are two important periods during development in which a surge of testosterone has been reported to defeminize and masculinize the brain of male rodents: late gestation and shortly after birth (Weisz and Ward, 1980). Whereas many studies have examined the sexual differentiation of reproductive components of the brain, much less has been published regarding the organizational differentiation of the HPA axis. Postnatal gonadectomy of male rats has been reported to cause a more female-like HPA axis activity in adulthood, characterized by increased basal and stress-induced corticosterone secretion (Patchev et al., 1995). Further, inhibiting the aromatase enzyme to prevent the conversion of testosterone to estradiol in neonatal males, has similar lasting consequences of increased basal and stress-induced corticosterone in adulthood. These results implicate the actions of estradiol, as the result of the aromatization of testosterone in the male on the organization of sex differences in the HPA axis (Lucion et al., 1996; de Kloet et al., 1998). The organizational actions of perinatal steroids have been further supported by studies examining the pulsatile patterns of corticosterone secretion throughout the day, where males show a lower amplitude and frequency of corticosteroid pulses compared to females (Seale et al., 2005a). Administration of an AR antagonist in a perinatal male increases the amplitude and frequency of corticosteroid pulses to resemble that of adult females. Moreover, perinatal gonadectomy of males also leads to a female-like pulsatile pattern in adulthood, while a single dose of testosterone following gonadectomy reverses this effect. Moreover, females treated with testosterone within 24 h of birth show a male-like pattern of corticosterone secretion (Seale et al., 2005a,b). These studies implicate an organizational action of testosterone directly through AR or indirectly through ER, *via* the aromatization of testosterone to estradiol, on the HPA axis (Seale et al., 2005a,b).

### Development of the HPA Axis During Early Life

During pregnancy, the stress response of the fetus is immature and relies heavily on inputs from the maternal and placental systems (Gunn et al., 2013). During late gestation, the fetus becomes capable of secreting CRH and ACTH in response to maternal stress, resulting in corticosterone production (Gunn et al., 2013). Basal levels of corticosterone during this time are similar to those of adults (Meaney et al., 1985b), suggesting functional HPA axis activity. From PND4 to PND14, basal corticosteroid levels drop, accompanied by substantially decreased ACTH and corticosterone production in response to stress (Schmidt et al., 2003). This period is known as the “stress hypo-responsive period” (SHRP; Buschdorf and Meaney, 2016). During this time, the expression of GR and MR mRNA is significantly increased (Yi et al., 1994). Accompanied by the lower levels of corticosterone, these changes are thought to dampen the HPA axis responses. Stress exposure during the SHRP induces a slight increase of expression of c-Fos mRNA in the PVN but does not influence ACTH or peripheral corticosterone secretion (Levine, 1994). Such data suggests an adrenal insensitivity may also play a role in the SHRP, but further mechanisms have not yet been determined.

Recent studies show that SHRP can be maintained through the influence of maternal care (Buschdorf and Meaney, 2016). Maternal care, quantified through observations of pup licking and maternal arch-backed nursing, are highly correlated with each other (Caldji et al., 1998). Dams categorized by levels of maternal care show a causal relationship to epigenetic reprogramming that alters negative feedback sensitivity through changes in DNA methylation and histone modifications (Weaver et al., 2004; Buschdorf and Meaney, 2016). Several studies suggest this may be related to the transcription factor, nerve growth factor-inducible protein A (NGFI-A) which binds to a promoter region on exon 1–7 of *GR* (Caldji et al., 1998) consisting of two CpG dinucleotides on opposing ends of the response element that are methylated on the day of birth. Offspring that received high maternal care show higher amounts of demethylation of 5′ CpG sites in the NGFI-A binding region in adulthood (Buschdorf and Meaney, 2016). Offspring that received low levels of maternal care displayed no change in methylation. Point mutation studies indicate that the NGFI-A binding strength and gene expression is determined by the presence of a methyl group at the 5′ CpG site, where a mutated 5′ CpG site resulted in increased transcription of *NR3C1*, the gene encoding GR (Weaver et al., 2004). These studies support the actions of low maternal care to program increased GR expression and increase feedback sensitivity of adult offspring (Liu et al., 1997). Additional studies show decreased corticosterone and ACTH responses to acute stress in adulthood of high maternal care-exposed offspring. Such data further support epigenetic reprogramming of the 5′ CpG site. A genome analysis of chromosome 18 containing *NR3C1* found that varying amounts of maternal care correlated with changes in protocadherin loci (McGowan et al., 2011) which regulate the development of the CNS (McGowan et al., 2011) thereby implicating maternal care during the SHRP for proper brain development. However, it is important to note that the promoter region on exon 1–7 of *GR* only accounts for about 1% of all GR mRNA transcripts in the hippocampus. Reports examining promoter region on exon 1–7 of *GR* methylation found that upregulation of NGFI-A did not alter stress-induced activation of the promoter region on exon 1–7 of *GR* transcription or total expression of GR (Makino et al., 1995). Consistent with this, Witzmann et al. (2012) showed low methylation levels at the 5′ CpG site following acute stress, further indicating that NGFI may not drive the promoter region on exon 1–7 of *GR* transcription nor play a role during the acute stress response (Witzmann et al., 2012). Thus, while epigenetic reprogramming is altered through maternal care, specific mechanisms in which this occurs are still unclear.

Maternal separation during fetal development is another variable that influences HPA axis development and adult patterns of stress-reactivity (Boccia and Pederson, 2001). Prolonged separation from the dam is associated with a hyperactive HPA axis and increased anxiety- and depressive-like behaviors in adult offspring (Liu et al., 1997). In contrast, brief separation increases maternal attentiveness to pups, resulting in better attenuation of the stress-response (Boccia and Pederson, 2001). Rodent studies have demonstrated that these changes are partially a consequence of alterations in the dopaminergic system since prolonged maternal separation caused decreased dopamine uptake associated with changes in dopamine transporter expression (Curley et al., 2011). This is thought to lead to increased stress-induced dopamine activity resulting in a hyperactive HPA axis. 5-HT signaling has also been shown to be altered by maternal separation with decreased metabolism in the AMY and enhanced concentrations of 5-HT and associated metabolites in the dorsal raphe nucleus and cingulate cortex (Arborelius and Eklund, 2007). Long-term consequences include the altered function of 5-HT receptors and transporters, as well as decreased expression of 5-HT receptor subtypes in the PFC and hypothalamus (Ladd et al., 2005). These changes correlate with increased anxiety- and depressive-like behaviors, suggesting that a signaling pathway linking the dopaminergic and serotonergic systems with stress responses exists, however, specific mechanisms have yet to be elucidated (Zakharova et al., 2009).

Paternal influences on the stress axis of adult mice have also been reported. Males exposed to 6 weeks of chronic variable stress before breeding had offspring of both sexes with reduced HPA axis activation to acute restraint in adulthood (Rodgers et al., 2013). This correlated with gene transcription changes in the PVN and BNST of offspring suggesting the possibility of epigenetic reprogramming through the male lineage. Studies have also investigated paternal retrieval and grooming effects in offspring. Testosterone levels were decreased in offspring from rats with increased paternal retrieval, as was AVP expression in the BNST, and this correlated with reduced aggressiveness in social interaction tests, such as the resident-intruder test (Frazier et al., 2006). Such data suggests an important hormonal link between paternal care, testosterone levels, and aggression. AVP immunoreactivity in the PVN was also found to be increased with reduced paternal care (Rodgers et al., 2013), correlating with increased stress-induced corticosterone secretion, further linking paternal care and HPA axis responses. Nonetheless, specific mechanisms of how paternal transmission to the offspring occurs have yet to be elucidated.

### The Development of the HPA Axis at Puberty

Puberty is a unique developmental event, influenced largely by the maturation of the hypothalamic-pituitary-gonadal (HPG) axis, which is responsible for gonadal maturation and adult hormone secretory patterns (Ojeda and Urbanski, 1994). Some reports also suggest that this represents a second critical period for organizational actions of gonadal hormones that further sculpt the HPA axis into its adult-like characteristics (Romeo, 2003; see review Romeo, 2010 for a more thorough analysis of age-dependent changes in the HPA axis).

Importantly, HPA axis reactivity is significantly greater before puberty than after puberty. Rat studies in males show an increased and prolonged stress-responsive release of ACTH and GC prepubertally in comparison to post-pubertal animals (Goldman et al., 1973; Romeo et al., 2004a). Similarly, the stress-induced activity of CRH neurons in the prepubertal PVN is greater than that of adults, demonstrating that the prolonged prepubertal pattern of corticosterone and ACTH may be driven by increased hypothalamic CRH synthesis (Romeo et al., 2004b; Viau et al., 2005) and altered by the onset of male puberty. These findings indicate a blunted GC-dependent negative feedback in prepubertal males (Romeo et al., 2004a).

Studies in pre-pubertal male rodents show elevated HPA activation, with increases in CRH activation following restraint in comparison to adults, indicating that PVN CRH neuron activity changes across puberty (Romeo et al., 2006). Following a corticosterone injection, Romeo and McEwen (2006) showed increased GR expression in regions of the brain, such as HC, AMY, and PFC, in adolescents compared to adults. This observation further indicated that puberty represents a critical period during development that renders the brain more vulnerable to environmental perturbations and increases the risk of HPA-related neuropathologies (Romeo and McEwen, 2006). The changes in the HPA axis do not appear to be the consequence of pubertal rises in testosterone (Romeo, 2003). However, because the initial increase of gonadotropin-releasing hormone (GnRH) secretion and kisspeptin occurs near the onset of puberty, one possibility is that changes in the HPA axis observed across puberty are preprogrammed developmental events that are independent of changes in gonadal hormones (Romeo, 2003).

Some reports further suggest that the pubertal rise in estradiol may also play a role in shaping the adult HPA axis. Studies in pre-pubertal females show an inhibitory effect of estradiol on stress-induced HPA axis function, while estradiol treatment in post-pubertal females shows a stimulatory effect of estradiol during the acute stress response (Evuarherhe et al., 2009). Further, regardless of whether females were ovariectomized before or after puberty, administration of estradiol consistently elevated basal and stress-induced GC secretion, as well as GC pulse amplitude and frequency (Evuarherhe et al., 2009). Data suggests there is a reversal effect of estradiol on HPA axis function during puberty where estradiol is inhibitory before puberty and stimulatory post-puberty, implying an estradiol-independent mechanism in the development of the HPA axis during puberty in adult females (Evuarherhe et al., 2009).

### Disruption of the Maternal-Fetal HPA Axis and Adult Disease Risk

A growing body of studies has described fetal risk factors for adult diseases that form the basis for the hypothesis of the Developmental Origins of Health and Disease (DOHaD; Sandman et al., 2015). The DOHaD postulates that there is a critical period of development where the fetus is most sensitive to certain environmental influences that significantly impact short- and long-term health (Harris and Seckl, 2011). Such environmental influences include maternal stress, which is a likely correlate of fetal overexposure to GC, implying a common pathway in which environmental insults become linked between mother and fetus (Edwards et al., 1993; Cottrell and Seckl, 2009).

Commonly, administration of synthetic GCs has been a common clinical treatment for women at risk for preterm labor, to improve survival of the newborn by allowing proper lung maturation (Liggins and Howie, 1972; Crowther et al., 2019). Betamethasone or DEX is often used (Roberts and Dalziel, 2006) in the clinic and they do not appear to differ in efficacy (Crowther et al., 2019). DEX has also been used to treat female fetuses diagnosed with congenital adrenal hyperplasia (CAH; Speiser et al., 2010), an autosomal recessive disorder that results in the deficiency of 21-hydroxylase that impairs the synthesis of corticosterone and causes impaired synthesis of adrenal enzymes (Speiser et al., 2010). Therefore, steroid metabolism is redirected to adrenal androgens, resulting in abnormal masculinization of genital development and behaviors in females. Consequently, dexamethasone is administered to female CAH fetuses to inhibit adrenal androgen production and minimize these effects. Notably, these females represent a population that is exposed to dexamethasone early in gestation, a critical period for fetal development. More recent studies have begun to elucidate long- and short- term consequences of prenatal exposure to excess GC, causing programming effects in the HPA axis of the fetus that result in dysregulation of important physiological functions in adulthood.

**Figure 3:**
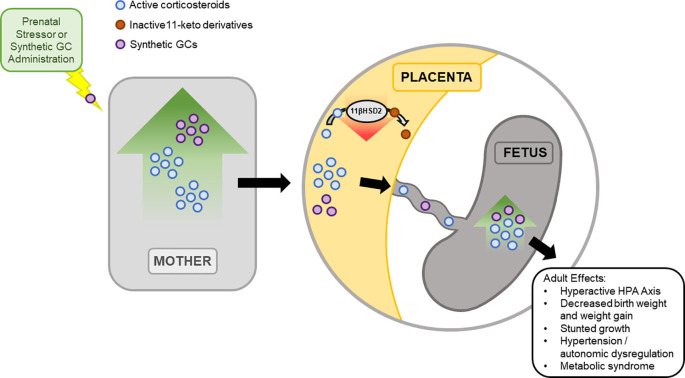
Prenatal exposure to excess GCs has short- and long- term effects. During pregnancy, 11-β hydroxysteroid dehydrogenase type 2 (11β-HSD2) expression in the placenta mediates the fetus’ exposure to excess maternal GC. 11β-HSD2 regulates the level of active GCs through the oxidation of maternal corticosteroids into inactive 11-keto derivatives, reducing fetal exposure to active GCs. While 11β-HSD2 concentration in the placenta is high during most of gestation, 11β-HSD2 levels drop during late gestation. As a result, fetal exposure to maternal GCs increases, leading to short term effects, observed immediately at birth into adulthood. To note, synthetic GC does not act as a substrate for 11β-HSD2 and bypasses this system to the fetus.

A key player mediating the consequences of maternal elevations of GC from stress is the placenta. During pregnancy, GC concentrations in maternal blood are higher than those of the fetus. This is a result of 11β-Hydroxysteroid dehydrogenase type 2 (11β-HSD2) expression by the placenta (Stroud et al., 2016). 11β-HSD2 oxidizes maternal corticosteroids into its inactive 11-keto derivatives (Benediktsson et al., 1997; Holmes et al., 2006), which buffers the increased levels of maternal GCs to the fetus. Levels of 11β-HSD2 are known to increase during early and mid-gestation, followed by a drop in late gestation to allow GC mediated fetal lung maturation. Furthermore, rodent studies suggest that the drop in 11β-HSD2 during late gestation allows increased transport of maternal GC to the fetus, increasing the vulnerability of the fetus during this period to unwanted programming effects (Edwards et al., 1993). Inhibition of 11β-HSD2 with carbenoxolone during late gestation shows effects similar to excess GC, further suggesting the importance of 11β-HSD2 in protecting the fetus from overexposure of GC (Brown et al., 1993; Lindsay et al., 1996; Holmes et al., 2006). Importantly, the administration of synthetic GCs bypasses the 11β-HSD system of the placenta since synthetic GCs are not a substrate for the enzyme (Figure 3; Low et al., 1994; Walker et al., 1994).

The short- and long- term effects on the fetus of maternal elevations of GC due to prenatal stress or prenatal exposure to synthetic GC have been found to depend on the length of exposure and time during development at which the insult occurs (Barbazanges et al., 1996). Fujioka et al. (1999) highlighted duration-dependent effects in rodents exposed to excess GCs in CRH-expressing PVN neurons, where chronic prenatal stress caused degeneration and apoptosis in these neurons (Baquedano et al., 2011). However, brief prenatal stress did not cause a change in CRH-expressing PVN neurons, suggesting that the duration of the stressor is important for impacting the normal development of PVN neurons (Fujioka et al., 1999) and perhaps neurons controlling the HPA axis (Insel et al., 1988).

Other studies have investigated the time-dependent effects of fetal exposure to excess GC. In rodents, *in utero* exposure to elevated GCs during late gestation has been shown to cause hyperactivity of the HPA axis in adulthood with elevated levels of basal GC and ACTH and decreased CRH expression (Kapoor et al., 2006), perhaps suggesting reduced negative feedback. Increased peaks and prolonged secretion of these hormones in response to stressors have also been observed (Muneoka et al., 1997; Shoener et al., 2006). In contrast, Kamphuis et al. (2002) demonstrated that postnatal exposure to a synthetic GC reduced stress-induced HPA activity in adulthood, further suggesting that the timing of GC elevation plays a crucial role in the developing stress response. Importantly, studies have also shown that treatment with allopregnanolone and 3β-diol can reverse the hyperactive HPA response to immune challenges in adult rats that were prenatally stressed (Brunton et al., 2015). This raises the possibility that the HPA axis can retain sufficient plasticity postnatally to allow reversal of maternal HPA axis hyperactivity in the offspring.

Offspring of rodent dams prenatally treated with GC during late gestation typically exhibit reduced birth weights that were consistently reduced throughout life compared to control offspring (Carbone et al., 2012). Reductions in birth weight have been suggested to be a direct consequence of a lack of placental 11β-HSD2 activity and excessive GC exposure on the fetus (Holmes et al., 2006), rather than due to malnutrition or poor maternal care (Nyirenda et al., 2001). Carbone et al. (2012) further observed reduced long bone lengths through PND21 in female, but not male, offspring prenatally exposed to DEX. This was thought to be linked to a dysregulation in growth hormone signaling, thereby reducing transcription of the *Igf-1* gene (Carbone et al., 2012). Similarly, *Ghrh* mRNA transcripts in the ARC of female, but not male, offspring were reduced (Carbone et al., 2012). Together, such data suggest a sex-specific mechanism in which prenatal GC exposure causes a reduction in *Ghrh to* decrease circulating GH, resulting in lower plasma IGF-1, and reduced birth weights. Evidence for molecular mechanisms underlying these events remains to be determined.

It is well-established that prenatal insults can impact the autonomic system in adult offspring exposed to fetal GC. Such long-term consequences include cardiovascular function (O’Regan et al., 2008). Angiotensinogen is an important component of the renin-angiotensin-system (RAS) that is upregulated by GCs (Tamura et al., 1995). Estrogens are found to have inhibitory influences over angiotensinogen gene expression (Dzau and Hermann, 1982), implying sex-dependent programming effects could be correlated with increased sensitivity to GC-mediated hypertension in females (O’Regan et al., 2004). Moreover, adult female offspring exposed to prenatal stress display a more prolonged increase in systolic blood pressure and a longer recovery period when subjected to restraint stress than males (Igosheva et al., 2007). Such data are consistent with recent observations of altered R-R interval variability (Heart rate variability; HRV) in adult offspring exposed to elevated fetal GCs. Adult female, but not male offspring of prenatal GC treated dams exhibit a reduction in high-frequency power when compared to control (Madhavpeddi et al., 2020). Because high-frequency power represents parasympathetic activity (Akselrod et al., 1981), these effects suggest that prenatal GC treatment programs the parasympathetic nervous system, which, in female offspring, is responsible for altered pressor and tachycardic responses. Whether this is the same as the responses of prenatal stress remains to be determined but implicate sex-specific programming effects of the ANS due to excess prenatal GC exposure.

In addition to alterations in ANS and neuroendocrine function in adult offspring exposed to elevated GC levels in development, the risk for adult metabolic dysfunction is also increased. Female rats prenatally exposed to high levels of GCs are hyperinsulinemic after oral glucose administration, with alterations in the expression of genes that mediate GC and lipid metabolism in subcutaneous fat (Brunton et al., 2013). By contrast, prenatal GC exposure was found to cause hyperglycemia following oral glucose in male offspring (Nyirenda et al., 1998), with alterations in the expression of genes that mediate GC and lipid metabolism in skeletal muscle and liver. These observations suggest sex-specific mechanisms where fetal exposure to GCs programs the stress response, leading to a dysregulation of glucose-insulin homeostasis (Brunton et al., 2013) and increased risk for diabetes mellitus type 2 in adulthood (Nyirenda et al., 1998). Moreover, prenatal exposure to GC alters hepatic gene expression, with decreased mRNA transcripts encoding *Pparα* and *Pgc1α*, key regulators of lipid and energy metabolism, and an increase of plasma triglyceride concentrations in offspring (Drake et al., 2010; Brunton et al., 2013). These observations suggest a mechanism in which fetal GCs affect important genes in fatty acid metabolism and increase the risk for hepatic steatosis in adulthood (Carbone et al., 2012).

## Future Studies on DOHaD

The developmental origins of the disease model posit that events during fetal and early-life correlate with long-term consequences that encompass the development of neuroendocrine signaling and ultimately susceptibility to neuropsychological and neuropathological diseases in adulthood. Improper development of the HPA axis is commonly suggested as the primary neuroendocrine system affected by alterations in the prenatal environment.

A growing body of evidence further suggests the placenta’s important role in mediating maternal-fetal interactions during the prenatal period. Placental hormones and cytokines are thought to regulate the effects of maternal stress on the fetal HPA axis, but it is important to consider the nature and timing of prenatal insults, as evidence suggests exposure to prenatal stress during various gestational periods and varying lengths of time exerts different effects on HPA axis activity (Sandman et al., 2015). However, precise mechanisms in which prenatal stressors influence neuroendocrine signaling between the maternal-placental-fetal interface are still unclear. In humans, a potential prospect is placental CRH, which is upregulated by maternal and fetal cortisol. Studies implicate placental CRH to directly mediate fetal pituitary-adrenal growth and steroid production, factors that are known to significantly affect HPA axis function during development and in adulthood (Green et al., 2016). Notably, more recent evidence examines the effects of fetal sex in placental crosstalk. In rodents, placentas of female fetuses show greater expression of genes associated with immune and endocrine function, while placentas of male fetuses tend to express more genes involved in inflammation (Cvitic et al., 2013). Moreover, the inherent sex differences in fetal metabolic needs and timing of GC administration related to maturation and decline of placental function towards the end of gestation could help us understand mechanisms by which fetal sex influences the maternal-placental-fetal interface, a primary regulator of the development of the HPA axis (Lassance et al., 2015; Sun et al., 2020).

Although there is a long history of research behind the HPA axis and development, much remains to be revealed. It is well known the HPA axis holds a fundamental role in maintaining proper neuroendocrine function and a large body of research strongly correlates the dysregulation of the HPA axis to neuropsychological and physiological disease risk. Therefore, it is reasonable to suggest a strong association between the development of the HPA axis to such diseases, emphasizing the importance of future developmental studies to address this large gap in our current knowledge.

## Author Contributions

RH and TH conceived of topic. RH wrote original outline. JS, NB, and MR wrote original manuscript. SM conceived of and constructed figures. AB proofread the manuscript and updated references. RH and TH edited and performed final proofreading. All authors contributed to the article and approved the submitted version.

## Conflict of Interest

The authors declare that the research was conducted in the absence of any commercial or financial relationships that could be construed as a potential conflict of interest.

## Abbreviations

11β-HSD2: 11-β hydroxysteroid dehydrogenase type 2
5-HT: Serotonin
ACTH: Adrenocorticotropic hormone
AMY: Amygdala
ANS: Autonomic nervous system
AR: Androgen receptor
ARC: Arcuate n.
ARNT2: Aryl hydrocarbon receptor nuclear translocator 2
AVP: Arginine Vasopressin
BNST: Bed n. of the stria terminalis
CAH: Congenital Adrenal Hyperplasia
cAMP: cyclic adenosine monophosphate
CBG: Corticosteroid-binding globulin
CeA: Central n. of the amygdala
CNS: Central nervous system
CRH: Corticotropin releasing hormone
CRH-BP: Corticotropin releasing hormone binding protein
DEX: Dexamethasone
DHEA: Dehydroepiandrosterone
DOHaD: Developmental Origins of Health and Disease
ER: Estrogen Receptor
ERE: Estrogen response element
ERα: Estrogen receptor alpha
ERβ: Estrogen receptor beta
FSH: Follicle stimulating hormone
GC: Glucocorticoid
GH: Growth hormone
GR: Glucocorticoid receptor
GnRH: Gonadotropin-Releasing Hormone
HC: Hippocampus
HPA: Hypothalamic-pituitary-adrenal
HPG: Hypothalamic-pituitary-gonadal
LH: Luteinizing hormone
MeA: Medial Amygdala
MPOA: Medial Preoptic Area
MR: Mineralocorticoid receptor
MSH: Melanocyte stimulating hormone
NGFI-A: Nerve growth factor-inducible protein A
NTS: Nucleus of Solitary Tract
OT: Oxytocin
PFC: Prefrontal Cortex
PND: Postnatal day
POMC: Proopiomelanocortin
PVN: Paraventricular nucleus
SCN: Suprachiasmatic nucleus
SHRP: Stress hypo-responsive period
SON: Supraoptic nucleus
TRH: Thyrotropin releasing hormone
TSH: Thyroid stimulating hormone
VMN: Ventromedial n.
